# Effect of Atmospheric Conditions on Pathogenic Phenotypes of *Arcobacter butzleri*

**DOI:** 10.3390/microorganisms10122409

**Published:** 2022-12-06

**Authors:** Rodrigo Martins, Cristiana Mateus, Fernanda Domingues, Roland Bücker, Mónica Oleastro, Susana Ferreira

**Affiliations:** 1 CICS-UBI-Health Sciences Research Centre, University of Beira Interior, 6200-056 Covilhã, Portugal; 2 Clinical Physiology/Nutritional Medicine, Medical Department of Gastroenterology, Infectious Diseases, Rheumatology, Charité–Universitätsmedizin Berlin, 12203 Berlin, Germany; 3 National Reference Laboratory for Gastrointestinal Infections, Department of Infectious Diseases, National Institute of Health Dr. Ricardo Jorge, Av. Padre Cruz, 1649-016 Lisbon, Portugal

**Keywords:** *Aliarcobacter butzleri*, virulence, stress response, atmospheric conditions

## Abstract

*Arcobacter butzleri* is an emergent gram-negative enteropathogenic bacterium widespread in different environments and hosts. During the colonization of the gastrointestinal tract, bacteria face a variety of environmental conditions to successfully establish infection in a new host. One of these challenges is the fluctuation of oxygen concentrations encountered not only throughout the host gastrointestinal tract and defences but also in the food industry. Oxygen fluctuations can lead to modulations in the virulence of the bacterium and possibly increase its pathogenic potential. In this sense, eight human isolates of *A. butzleri* were studied to evaluate the effects of microaerobic and aerobic atmospheric conditions in stressful host conditions, such as oxidative stress, acid survival, and human serum survival. In addition, the effects on the modulation of virulence traits, such as haemolytic activity, bacterial motility, biofilm formation ability, and adhesion and invasion of the Caco-2 cell line, were also investigated. Overall, aerobic conditions negatively affected the susceptibility to oxygen reactive species and biofilm formation ability but improved the isolates’ haemolytic ability and motility while other traits showed an isolate-dependent response. In summary, this work demonstrates for the first time that oxygen levels can modulate the potential pathogenicity of *A. butzleri*, although the response to stressful conditions was very heterogeneous among different strains.

## 1. Introduction

*Arcobacter butzleri* belongs to the novel *Arcobacteraceae* family and the *Campylobacterales* order. These bacteria are characterized as small, curved rods capable of motility by a single polar flagellum and are able to grow at temperatures from 15 °C to 42 °C [1,2]. Although *A. butzleri* has been described as microaerophilic, it can also grow under aerobic conditions [2,3]. This species is widespread in different environments and hosts, being isolated from different sources, such as animals, water, food, and human samples. The most likely route of transmission of this bacterium to humans is considered the consumption of contaminated food or water [4]. Infections with this species are often associated with bacteraemia, enteritis, watery diarrhoea, abdominal cramps, nausea, and vomiting [5]. *A. butzleri* was demonstrated to be the most prevalent species of its genus and it has been considered the fourth most frequent *Campylobacter-like* organism found in human diarrhoeic samples, also ranking the fourth most common pathogen isolated from faecal samples from individuals with acute enteric disease [6,7,8,9]. In fact, *A. butzleri* has been classified by the International Commission on Microbiological Specification for Food as a moderate hazard to humans [10]. The pathogenic potential of *A. butzleri* has already been evidenced by its ability to adhere, invade, and even survive intracellularly in intestinal epithelial cell lines as well as by its cytotoxic and barrier-breaking effects [5,11,12,13,14].

As an emergent enteropathogen, *A. butzleri* is exposed to a variety of stressful conditions when spreading to and colonizing new hosts. During its passage through the gastrointestinal tract, *A. butzleri* is exposed to conditions, such as shifts in pH, temperature, osmotic pressure, salinity, and nutrient depletion as well as the presence of harmful substances, namely bile, reactive oxygen, and nitrosative species [15,16,17]. In addition, in the environment, host, or during food processing and preservation, different oxygen conditions can be found, which can play a crucial role in the transmission and spread of the bacterium or protect the bacterium when exposed to multiple stresses during its life cycle [18,19,20]. When the bacterium is exposed to different levels of atmospheric oxygen, its pathogenicity may be affected, and changes in the phenotype and stress-related mechanisms may occur [19,20,21]. 

With this work, we aimed to elucidate the role of aerobic and microaerobic atmospheric conditions on the physiological and virulence characteristics of several strains of *A. butzleri* isolated from human disease cases.

## 2. Materials and Methods

### 2.1. Bacterial Strain and Growth Conditions

Eight isolates of *A. butzleri* from human faecal samples isolated as part of the Portuguese surveillance program of campylobacteriosis conducted by the National Institute of Health Dr Ricardo Jorge were used in this study (INSA_AB#8, INSA_2680, INSA_2808, INSA_2999, INSA_3202, INSA_3711, INSA_3800, INSA_4015). Considering the stool cultures performed for *Campylobacter* sp, *Salmonella* sp, *Shigella* sp, and pathogenic *E. coli*, no co-isolation of another enteric pathogen was observed. The strains were first identified by the multiplex PCR described by Houf et al. (2000) followed by the confirmation of species by MALDI-TOF MS [22]. The strains were preserved at −80 °C in brain heart infusion medium containing 20% of glycerol (*v*/*v*). 

All the strains were inoculated in tryptic soy agar (TSA, VWR, Leuven, Belgium) and incubated at 37 °C for 24 h under aerobic or microaerobic (6% O_2_, ±7.1% CO_2_ and 3.6% H_2_) conditions previously to each assay. Then, overnight cultures in 10 mL of tryptic soy broth (TSB, VWR, Leuven, Belgium) were prepared and incubated in an orbital shaker at 100 rpm at 37 °C under aerobic or microaerobic conditions for use in the following assays, being this a modification applied to each assay.

### 2.2. Oxidative Stress

The susceptibility to oxidative stress was determined by the disc diffusion assay, as described in Kern et al. (2011), with a slight alteration in the standardization of the inoculum [23]. Sterile cellulose filter discs of 6 mm were loaded with 5 μL of 10% of hydrogen peroxide (H_2_O_2_) (LABKEM, Barcelona, Spain) or 250 mM methyl viologen (Sigma-Aldrich, St. Louis, MO, USA) and added to the centre of a TSA plate swabbed with a cellular suspension with ~10^8^ colony-forming units per millilitre (CFU/mL). After incubation at 37 °C in aerobic or microaerobic atmosphere for 48 h, the halo of inhibition was measured. This assay was performed at least three times, independently, for each strain.

### 2.3. Acidic Stress Survival

The survival to acidic stress was performed as described by Isohanni et al. (2013) [24]. Bacteria were recovered from an overnight culture under the test conditions by centrifugation for 5 min at 12,000× *g*. Then, after resuspension in TSB medium with pH of 4 and adjusting the final number of cells to about 10^7^ CFU/mL, bacteria were incubated at 37 °C. The viable counts of the isolates were determined at 0, 20, 40, and 60 min of exposure to acidic pH by drop-plate method in phosphate-buffered saline (PBS) (Lonza, Walkersville, USA). The plates were incubated for 48 h at 37 °C under aerobic and microaerobic conditions, and CFU/mL was counted. This experiment was performed at least three times independently for each strain.

### 2.4. Serum Survival

Serum bactericidal activity was evaluated according to the protocol described by Mateus et al. (2021) with slight modifications [25]. The serum was recovered from the blood of healthy volunteer donors, and after separation by centrifugation at 2000 rpm for 10 min at 4 °C, it was pooled and stored in aliquots at −80 °C in sterile cryogenic vials. For the experiment, the bacterium was recovered, washed, and resuspended in PBS at a final concentration of 10^8^ CFU/mL. In microtubes with 180 μL of pooled human serum, 20 μL of the bacterial suspension was added and incubated at 37 °C. As control, serum was replaced with PBS. Viable counts were performed at 0, 15, 30, 45, and 60 min after exposure to human serum. The plates were incubated for 48 h at 37 °C under aerobic or microaerobic conditions, and CFU/mL was counted. This assay was performed at least three times independently for each strain.

### 2.5. Haemolytic Activity

The haemolytic activity of the isolates was assessed as described in Ferreira et al. (2014) with an adjustment on the bacterial suspension concentration [7]. Erythrocytes were collected from one healthy donor, then washed three times with PBS, and a stock suspension was prepared in the same buffer. All the isolates were collected by centrifugation, washed with PBS, and the final bacterial suspension was adjusted to 10^9^ CFU/mL. In a 96-well plate with a U bottom (Thermo Scientific, Waltham, MA, USA), 100 μL of the bacterial suspension and 100 μL of 2% (*v*/*v*) erythrocytes solution was added. A negative control of PBS with the erythrocytes and a positive control of 1% (*v*/*v*) of Triton X-100 with erythrocytes were included. The plate was incubated at 37 °C for 18 h in aerobic or microaerobic conditions. After incubation, the plate was centrifuged at 1000× *g* for 5 min, and 100 μL of the supernatant from each well was removed to a 96-well plate (VWR, Belgium), and the absorbance was measured at 492 nm in a microplate reader (Biorad, xMark, Hercules, CA, USA). Each assay was performed with four replicates in at least three independent assays.

### 2.6. Motility and flaA Relative Expression Assays

The motility profile of the isolates was evaluated as described by Ferreira et al. (2018) with slight alterations, namely the bacterial suspension concentration and the incubation temperature [26]. Five μL of the bacterial suspension of ~10^8^ CFU/mL was inoculated by stabbing the centre of semisolid TSA (0.4% agar) plate. After 48 h of incubation at 37 °C in aerobic or microaerobic atmosphere, the motility halo was measured. This assay was performed at least three times independently for each strain.

The relative expression of the *flaA* gene was analysed by quantitative real-time PCR (RT-qPCR). The isolates of *A. butzleri* strains were grown until mid-exponential phase, recovered, washed, and RNA isolated using the TripleXtractor reagent (GRiSP, Porto, Portugal). Next, a treatment with DNase I was performed followed by the cDNA synthesis using a GRS cDNA Synthesis master mix (GRiSP, Portugal) according to the manufacturer’s instructions. RT-qPCR reaction mixture was performed in a total final volume of 10 µL, containing 5 µL of NZY qPCR Green master mix (2×) (NZYTech Ltd., Lisboa, Portugal), 0.4 µM of specific primers, and 1 µL of cDNA following the program: 2 min at 95 °C, 40 cycles for 5 s at 95 °C followed by 30 s at 60 °C, and at last 5 s in crescent gradient to 95 °C in a CFX Real-Time PCR System (Bio-Rad, USA). The primers used to perform the qRT-PCR were flaA_F and flaA_R targeting *flaA* gene [27], and P338_F and P518_R targeting 16S gene [28]. The relative expression was determined using the comparative threshold cycle (2^−∆∆CT^) method and the expression of the 16S rRNA gene was used for relative quantification. All qPCR reactions were performed in duplicates and three independent assays.

### 2.7. Biofilm Formation Ability

Biofilm formation ability of the isolates was assessed as described by Mateus et al. (2021) with some modifications [25]. In this case, 24-well polystyrene plaques were used instead of 96-well polystyrene plaques and, consequently, the volume added was also adjusted. Thus, plates of 24-well polystyrene (VWR, Belgium) were inoculated with 500 μL of a bacterial suspension with about 10^8^ CFU/well and incubated at 37 °C for 48 h in aerobic or microaerobic conditions. Following the incubation, the medium was removed, and the wells were dried for 1 h at 55 °C. Then, 500 μL of 0.1% (*w*/*v*) crystal violet was added for 15 min at room temperature. The unbound crystal violet (AMRESCO, Leuven, Belgium) was removed, and the wells were washed three times with distilled water and dried again for 15 min. Bound crystal violet was solubilized with a 30% methanol/10% acetic acid solution. Finally, the absorbance at 570 nm was recorded using a microplate reader (Biorad, xMark). Each assay was performed with four replicates in at least three independent assays.

### 2.8. Adhesion and Invasion of Caco-2 Cells Line

The Caco-2 human intestinal epithelial cells were maintained in Dulbecco’s modified Eagle medium (DMEM) (Sigma-Aldrich, USA) supplemented with 10% (*v*/*v*) fetal bovine serum (FBS) (PAN-Biotech, Aidenbach, Germany), 1% (*v*/*v*) nonessential amino acids (Lonza, USA), 100 μg/mL of streptomycin, and 100 U/mL of penicillin (Sigma-Aldrich, USA). The cells were grown in tissue culture flasks maintained at 37 °C in 5% CO_2_ and 95% air. Medium was replaced every two days until cells reached a semi-confluent state of about 80%. Then, the cells were seeded in 24-well polystyrene plates with 1 × 10^5^ cells/well and left to multiply for 48 h in the same conditions [25].

The adhesion and invasion assays were performed as described in Ferreira et al., (2014) with an adjustment on the bacterial concentration used for infection [7]. The isolates were collected by centrifugation, washed, and resuspended in the medium used for Caco-2 cell culture without antibiotic with a final concentration between 1.5–7 × 10^7^ CFU/mL. Then, Caco-2 cells were washed twice with PBS and 500 μL of each bacterial suspension was added to each well followed by incubation for 3 h. Following this period, the cells were washed three times with 500 μL of PBS and the number of interacting (adherent and internalized) bacteria was determined by lysing the Caco-2 cells with the addition of 500 μL Triton X-100 (Sigma-Aldrich, USA) at 1% (*v*/*v*) for 5 min followed by plate count of bacteria. For the study of bacterial invasion, 500 μL of medium containing 125 μg/mL of gentamicin (Sigma-Aldrich, USA) was added to each well for 1 h. Then, cells were washed three times with 500 μL of sterile PBS and lysed with the addition of 500 μL Triton X-100 at 1% (*v*/*v*) in each well for 5 min. The released intracellular bacteria were enumerated by plating serial dilutions of the lysates in TSA plates. Results were expressed as the variation of adherent (interacting-internalized bacteria) or internalized bacteria to the initial inoculum used in the assay for adhesion and invasion, respectively. In this assay, three replicates per experiment were used in at least three independent measurements.

### 2.9. Statistical Analysis

Data were presented with mean values ± standard deviation (SD) or error of the mean (SEM) according to the assay. Statistical analysis was done with GraphPad Prism (GraphPad Software version 8, San Diego, CA, USA). Student’s *t*-test and *t*-test with Holm–Sidak correction were used. *p* < 0.05 was considered statistically significant.

## 3. Results

### 3.1. Effect of Atmospheric Conditions on Arcobacter butzleri in Host Conditions of Stress

**Figure 1:**
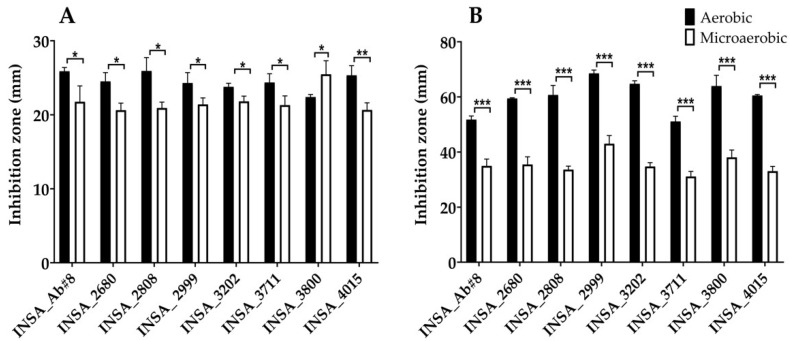
Susceptibility of eight clinical isolates of *Arcobacter butzleri* to oxidative stress when exposed to (**A**) 10% of hydrogen peroxide or (**B**) 125 mM of methyl viologen when grown in aerobic or microaerobic conditions. Results were analysed between atmospheric conditions using Student’s *t*-test. The data shown represent the mean ± standard deviation of at least three independent assays. * *p* < 0.05, ** *p* < 0.01, *** *p* < 0.001.

The effects of oxygen on the response of *A. butzleri* to the stressful conditions the bacterium may find in the host were analysed. Both the hydrogen peroxide (H_2_O_2_) capable of producing hydroxyl radicals and the superoxide generator methyl viologen [29] showed to inhibit all the eight bacterial isolates under the two testing atmospheric conditions (aerobic or microaerobic conditions). When exposed to H_2_O_2_, most of the isolates showed a significantly increased susceptibility under aerobic conditions with the exception of the isolate INSA_3800, which increased its susceptibility when grown in microaerophilic conditions (Figure 1A). The same trend was observed for exposure to methyl viologen (Figure 1B) with an even greater impact on the susceptibility.

**Figure 2:**
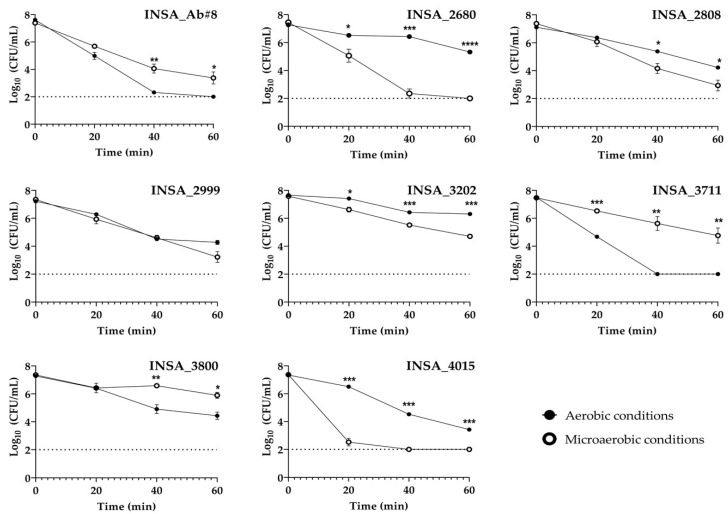
Effect of acidic stress in the survival of eight clinical isolates of *Arcobacter butzleri* when exposed to TSB medium at pH 4 while grown in aerobic and microaerobic conditions. The dashed line represents the detection limit of the assay. Results were analysed between atmospheric conditions using Student’s *t*-test. The data shown represent the mean ± standard deviation of at least three independent assays * *p* < 0.05, ** *p* < 0.01, *** *p* < 0.001, **** *p* < 0.0001.

Following the exposure of *A. butzleri* to a medium at pH 4, we can observe different profiles of susceptibility to acidic pH, with all the strains surviving at least twenty minutes under aerobic or microaerobic conditions (Figure 2). In particular, four of the isolates presented a higher capacity of survival to acid stress in aerobic conditions, three isolates in microaerobic conditions, and one isolate did not show a significant change.

**Figure 3:**
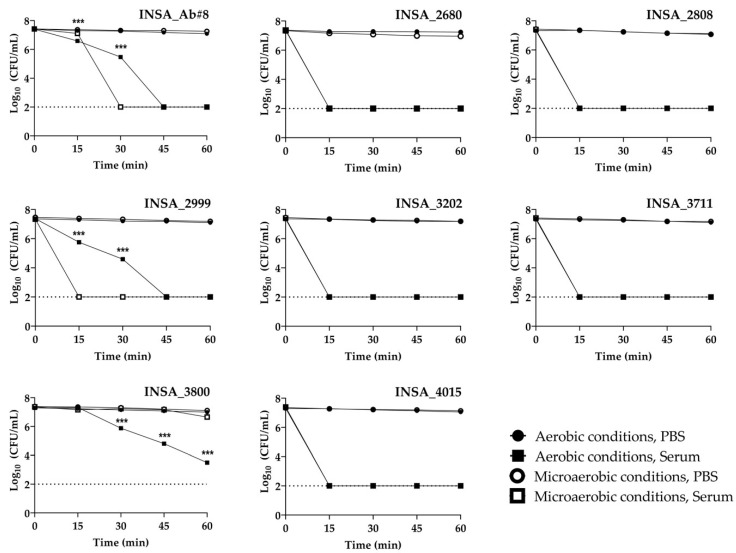
Survival of eight clinical isolates of *Arcobacter butzleri* to human serum when grown in aerobic or microaerobic conditions. The dashed line represents the detection limit of the assay. Results were analysed between atmospheric conditions when exposed to serum using Student’s *t*-test The data shown represent the mean ± standard deviation of at least three independent assays. *** *p* < 0.001.

When exposed to human, serum two distinct survival profiles were observed among the isolates studied (Figure 3). Five of the isolates were not capable of surviving after 15 min of exposure to human serum in both aerobic and microaerobic conditions. Although the isolates INSA_Ab#8 and INSA_2999 showed improved survivability to human serum when grown in aerobic conditions, in microaerobic conditions, the isolate INSA_Ab#8 was shown to be capable of surviving an additional 15 min when compared to the isolate INSA_2999. In turn, the isolate INSA_3800 was capable of surviving for 60 min in human serum when exposed to both conditions, with a decrease in survival after 30 min under aerobic conditions.

### 3.2. Effect of Atmospheric Conditions on Virulence Traits of Arcobacter butzleri

**Figure 4:**
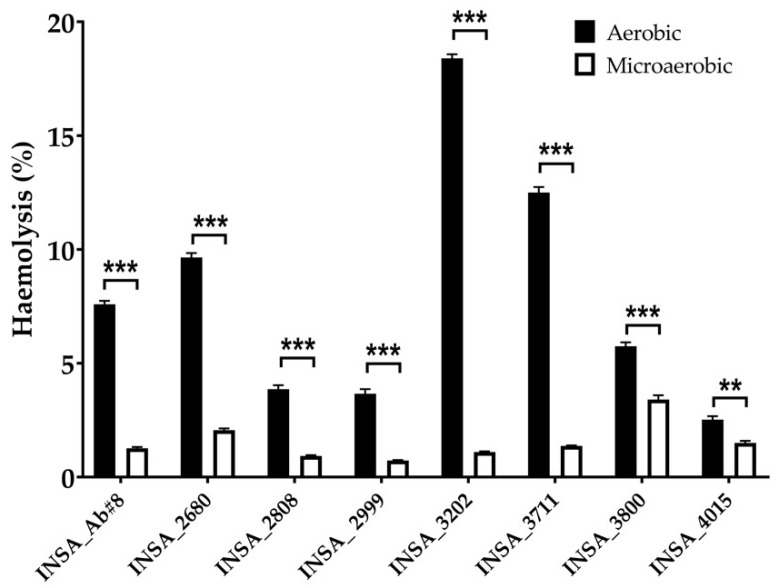
Haemolytic activity of eight clinical isolates of *Arcobacter butzleri* against human erythrocytes when in aerobic and microaerobic conditions. Results were analysed using Student’s *t*-test. The data shown represent the mean ± standard deviation of at least three independent assays. ** *p* < 0.01, *** *p* < 0.001.

To evaluate the impact of different levels of oxygen in the virulence of *A. butzleri*, we tested the effect of aerobic and microaerobic conditions in the virulence traits that may have relevance for the pathogenicity potential of the bacterium but also in its survival or dissemination in different environments. Regarding the haemolytic activity, it was higher under aerobic than under microaerobic conditions for all the tested isolates (Figure 4). It is also possible to note that the isolates showed a more diverse range of values of haemolytic activity when cultivated under aerobic versus microaerobic conditions, highlighting the strain-dependent phenotype of the isolates.

**Figure 5:**
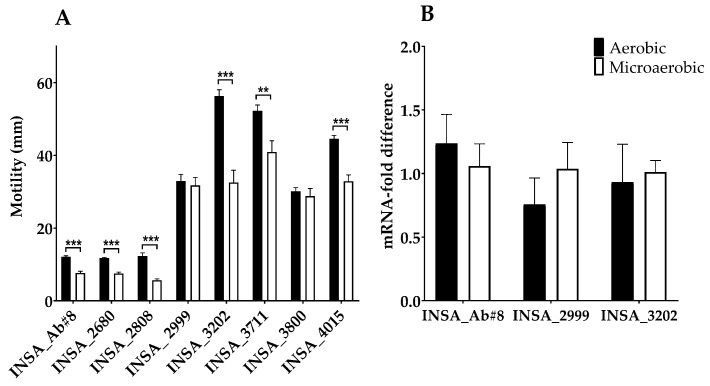
(**A**) Motility halo of eight clinical isolates of *Arcobacter butzleri* in millimetres when growth in aerobic or microaerobic conditions. (**B**) Comparison of the relative expression of the *flaA* by qPCR in *Arcobacter butzleri* INSA_Ab#8, INSA_2999 and INSA_3202 isolates when comparing aerobic versus microaerobic conditions. Results were analysed using Student’s *t*-test. The data shown represent the mean ± standard deviation of at least three independent assays. ** *p* < 0.01, *** *p* < 0.001.

Concerning the effect of atmospheric conditions on motility, when measuring the motility halos, a trend to higher motility under aerobic conditions compared to the microaerophilic growth was observed with exception of two of the isolates for which no difference was detected (INSA_2999 and INSA_3800) (Figure 5A). In line with the previous results, heterogeneity in the motility phenotypes among isolates was also observed, although high, intermediate, and low motility types could be distinguished.

Regarding the relative expression of the *flaA* gene, three isolates were selected according to different motility abilities (INSA_3202, high motility; INSA_2999, intermediate motility; INSA_AB#8, low motility). However, when the relative expression of the *flaA* was analysed, no significant differences were found for the assays performed under both conditions or among the isolates. In addition, for each strain, it was not possible to correlate motility with *flaA* expression for any of the atmospheric conditions. (Figure 5B).

**Figure 6:**
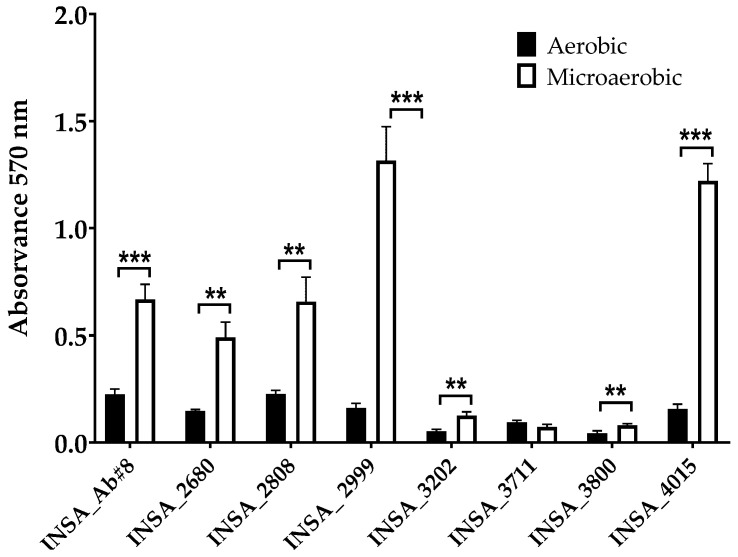
Biofilm formation ability by eight clinical isolates of *Arcobacter butzleri* evaluated by crystal violet staining when growth in aerobic and microaerobic conditions. Results were analysed using Student’s *t*-test. The data shown represent the mean ± standard deviation of at least three independent assays. ** *p* < 0.01, *** *p* < 0.001.

Concerning the biofilm formation ability, the majority of the isolates demonstrated to be able to form more biofilm when grown and incubated under microaerobic conditions (Figure 6) with only one isolate (INSA_3711) showing no significant difference when exposed to both atmospheric conditions. The diverse behaviour of the different strains in their ability to form biofilms under the experimental conditions carried out is also worth noting.

**Figure 7:**
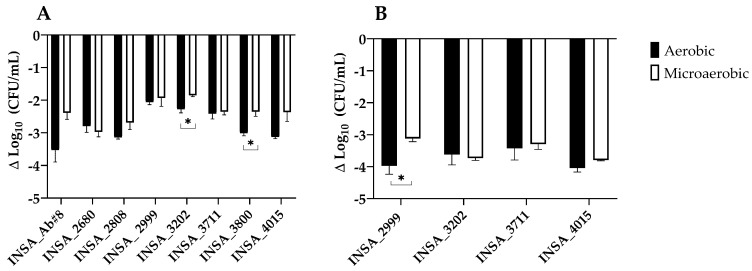
Adhesion (**A**) and invasion (**B**) of eight *Arcobacter butzleri* isolates to Caco-2 cells when grown under aerobic or microaerobic conditions. Strains not invading Caco-2 cells are not shown in the graphic. Results were analysed using Student’s *t*-test. The data shown represent the mean ± standard error of the means of at least three independent results. * *p* < 0.05.

Regarding the potentiation of the adhesion or invasion to Caco-2 cells after growth under both atmospheric conditions, few differences were observed (Figure 7). In the adhesion assay, atmospheric conditions did not have an influence on the majority of isolates except for two, INSA_3202 and INSA_3800, that showed a significant increase in adhesion ability in microaerobic conditions compared to aerobic growth (Figure 7A). Concerning the invasion ability, only half of the tested isolates (INSA_2999, INSA_3202, INSA_3711, and INSA_4015) showed to be able to invade Caco-2 cells; only isolate INSA_2999 showed a decreased invasion ability in aerobic conditions (Figure 7B).

## 4. Discussion

The wide distribution of *A. butzleri* exposes the bacterium to varying levels of oxygen in its different environments or hosts. In the food chain, it may find different conditions impact its transmission or dissemination or similarly in the host where the percentages of oxygen found in the bloodstream differ from those found in the gut. The observed bacterial response may be influenced by different degrees of oxygen, inducing changes in the regulation of stress responses and metabolic profiles and also modulating their virulence traits, which may influence their pathogenic potential [19,20,21]. Therefore, as bacteria must tolerate or adapt to extremely stressful conditions, in order to propagate, colonize new hosts, and therefore establish a successful infection [15,16,17], this study aimed at a characterization of the role of aerobic and microaerobic conditions on the survival of *A. butzleri* to stress and its virulence traits.

During the infection process, pathogens are exposed to several stress factors, such as oxidative stress, resulting from the production of reactive oxygen species (ROS) by the host immune system in order to control the infection through death or inhibition of the bacterial growth [15,20,30]. In the present study, we observed that the majority of the clinical isolates increased their susceptibility to both hydrogen peroxide and methyl viologen when grown under aerobic conditions. This can be due to a higher intracellular accumulation of these compounds under aerobic conditions, leading to impairment of essential mechanisms in the bacteria cell, such as lipid oxidization, diminished cell viability, and others [20]. In response to ROS, bacteria encode several detoxification enzymes, such as catalase and alkyl hydroperoxide reductase, to be able to survive the damaging effects of oxidative species [15]. Studies in *C. jejuni* showed that exposure to aerobic atmospheric resulted in an upregulation of several genes associated with oxidative protection, such as the catalase encoding gene (katA), superoxide dismutase (sodB), and peroxidase (ahpC) gene [20,31]. In *A. butzleri* homologues for some of these genes, namely the aphC and katG, were already described [32], and similar behaviour to that of *C. jejuni* may have a parallel in *A. butzleri* and deserves to be investigated. Regarding the response of *A. butzleri* to methyl viologen, it is similar to the resistance pattern described for *C. jejuni*, with both species encoding a single superoxide dismutase, SodB. This would suggest that *A. butzleri* can share a detoxification pathway of superoxide species analogous to the one described for *C. jejuni* [32,33,34].

A combination of mild acid and aerobic conditions can often be encountered by foodborne pathogens not only in the food preservation environment but also during passage through the gastrointestinal tract, namely the stomach [35,36]. In that niche, after ingestion, bacteria must survive a range of pH values from pH 6 to pH 2 when in a fasting state and a few minutes after intake of a meal, respectively [37]. These pH fluctuations can lead to damage to the outer bacterial membrane and disruption of cell homeostasis, ultimately leading to cell death. To avoid this, the bacterium needs to express acid defence mechanisms and be able to adapt and repair, improving their survival [15]. *A. butzleri* has been previously described as sensitive to low pH values [38], which is supported by the results obtained in the present study. It was also possible to observe that four of the eight isolates tested were more capable of resisting stress induced by acidic conditions when exposed to aerobic conditions versus microaerobic conditions while the remaining isolates presented the opposite behaviour. Although these mechanisms in *A. butzleri* are not fully described, a parallel to *C. jejuni* is plausible, for which a cross-protection effect of the exposure to aerobic conditions in the survival of that species under acid stress has been observed, mostly relying on the upregulation of heat shock proteins [39]. As also suggested by Murphy et al., (2006) for *C. jejuni*, the strains that are already more adapted to aerobic conditions may not utilize the same protective effect, implying a possible aerobic adaptation displayed by the isolates that present a greater survival in aerobiosis [40].

Infections caused by *A. butzleri* have also been associated with cases of bacteraemia, suggesting that some strains can pass the vascular barrier and cause systemic infections [5]. The bactericidal effect of human serum on *A. butzleri* has already been described, showing that this bacterium is highly susceptible to normal human serum, which may constitute an effective protective barrier against systemic invasion as observed in *C. jejuni* [41]. However, serum-resistant strains or immunocompromised hosts can lead to systemic infections, as already described for several *Campylobacter* species. [42,43,44] On the other hand, one of the tested *A. butzleri* strains showed resistance to human serum in microaerobic conditions, suggesting an enhanced potential to cause infections and inflammation of damaged tissues in the host [45]. Nonetheless, for five of the eight tested strains, the survival profile found was similar in both atmospheric conditions studied while two isolates showed a higher survival in aerobic atmospheric, and one isolate presented a higher survival in microaerophilic conditions. A similar experiment was performed in *E. coli,* and the researchers suggested that superoxide dismutases, sodA and sodB could be involved in the survival against human serum by the stabilization of the outer membrane of the bacteria [46]. In the genome of *A. butzleri*, the superoxide dismutase sodB was already described [32]. The altered regulation of these genes may have a role in the obtained response since its upregulation was detected in cases where bacteria experienced higher levels of oxygen [19,20]. Despite haemolysis virulence genes having been described in the genome of *A. butzleri*, it is considered to have low haemolytic activity [2,7]. Here, we found that aerobic conditions potentiate haemolytic activity, which may be associated with the hypothesis that high levels of oxygen may expose the surfaces of eukaryotic cells, making them more susceptible to perforation and membrane-damaging agents, such as bacterial haemolysis [47], which could, in general, explain the results obtained. However, further exploration of these interactions should be carried out.

As oxygen levels may modulate virulence traits [48,49], we further evaluated if virulence-related features, such as bacterial motility, biofilm formation, host cell colonization, and invasion, were modulated by different atmospheric conditions. Regarding bacterial motility, we found that for most strains, this trait was increased under aerobic conditions. This may be related to a potential promotion of dissemination, since pre-existing inflammation is believed to promote the dissemination of *A. butzleri* to other tissues [7,13]. This is in line with observations of *Campylobacter concisus* where motility was increased by the presence of higher levels of oxygen related to pre-inflammation that could act as a regulatory switch to survival, promoting its dissemination [50,51]. To further explore the finding, we evaluated *flaA* gene (major flagellin) expression levels since mutants lacking the *flaA* gene resulted in a non-flagellated and immotile bacterium [52]. When evaluating the expression of the *flaA* gene of the isolates, no differences were observed in either atmospheric conditions tested, in line with results previously described by Ho et al. (2008), suggesting that other factors may contribute to the higher levels of motility under aerobic conditions [52].

Biofilms are able to increase the survival of bacteria, having a protective effect against host defence mechanisms [53]. This bacterial ability has also been associated with an increase in the persistence of the pathogen in the food industry [54,55]. Similarly to those described by Ferreira et al. (2013), our results demonstrate a general increase in the ability of biofilm formation under microaerobic conditions when compared to aerobic conditions [56]. Šilha et al., (2021) suggested that *Arcobacter-like* species tend to prefer microaerophilic environments but are capable of forming biofilms in both microaerobic and aerobic conditions [57]. However, other studies have shown that aerobic conditions could favour biofilm formation for most of the studied strains, showing that a strain-dependent response could be responsible for the variability of the results [50,57,58,59,60,61].

Adhesion is a critical factor in bacterial pathogenicity; it is often related to the ability to establish host cell infection and tissue colonization, and is the pre-requisite to cell invasion [62,63]. To avoid the extracellular harsh environment and physical stress imposed by the host, several bacteria invade the target cells and can survive, replicate, and disseminate to other tissues and thus establish and maintain a successful infection [63,64]. The oxygen levels in the gut are generally associated with anaerobic or microaerobic environments; however, some areas adjacent to the mucosal surface have been shown to present higher levels of oxygen [48]. Potential modulation of the adhesion and invasion ability based on oxygen levels may be related to the ability of *A. butzleri* to thrive in these areas [65,66]. However, in the present study, only two of the eight tested isolates showed significant differences regarding the potentiation of adhesion in microaerobic conditions. In addition, for one isolate, the microaerophilic conditions seemed to benefit its adhesive and invasive ability. This favours the hypothesis that the mucus layer could provide a preferable environment for this species instead of undergoing the process of invasion, or that higher levels of oxygen could lead to a downregulation of genes involved in the invasion process, thus, needing further investigation [48,66,67,68]. Nonetheless, the observed response appeared to be strain specific.

## 5. Conclusions

In conclusion, this work showed that *A. butzleri* displays a wide variety of phenotypes regarding physiological and virulence-related features, corroborating the already described heterogeneity and likely explaining the diversity of environments where it can be found. In addition, it reports for the first time the influence of oxygen levels in the modulation of virulence- and pathogenicity-associated traits of this bacterium; the underlying mechanisms need to be further investigated.

## Data Availability

Data is contained within the text.

## References

[1] Pérez-Cataluña A., Salas-Massó N., Diéguez A.L., Balboa S., Lema A., Romalde J.L., Figueras M.J. (2018). Revisiting the Taxonomy of the Genus *Arcobacter*: Getting Order from the Chaos. Front. Microbiol..

[2] Vandamme P., Vancanneyt M., Pot B., Mels L., Hoste B., Dewettinck D., Vlaes L., van den Borre C., Higgins R., Hommez J. (1992). Polyphasic Taxonomic Study of the Emended Genus Arcobacter with *Arcobacter butzleri* Comb. Nov. and *Arcobacter skirrowii* sp. Nov., an Aerotolerant Bacterium Isolated from Veterinary Specimens. Int. J. Syst. Bacteriol..

[3] de Blackburn C.W., McClure P.J., de Blackburn C.W., McClure P.J. (2009). 20—*Campylobacter* and *Arcobacter*. Foodborne Pathogens.

[4] Ferreira S., Oleastro M., Domingues F. (2019). Current Insights on *Arcobacter butzleri* in Food Chain. Curr. Opin. Food Sci..

[5] Collado L., Figueras M.J. (2011). Taxonomy, Epidemiology, and Clinical Relevance of the Genus *Arcobacter*. Clin. Microbiol. Rev..

[6] Collado L., Gutiérrez M., González M., Fernández H. (2013). Assessment of the Prevalence and Diversity of Emergent Campylobacteria in Human Stool Samples Using a Combination of Traditional and Molecular Methods. Diagn. Microbiol. Infect. Dis..

[7] Ferreira S., Queiroz J.A., Oleastro M., Domingues F.C. (2014). Genotypic and Phenotypic Features of *Arcobacter butzleri* Pathogenicity. Microb. Pathog..

[8] Van den Abeele A.M., Vogelaers D., Van Hende J., Houf K. (2014). Prevalence of *Arcobacter* Species among Humans, Belgium, 2008–2013. Emerg. Infect. Dis..

[9] Vandenberg O., Dediste A., Houf K., Ibekwem S., Souayah H., Cadranel S., Douat N., Zissis G., Butzler J.P., Vandamme P. (2004). *Arcobacter* Species in Humans. Emerg. Infect. Dis..

[10] International Commission on Microbiological Specifications for Foods (2018). Microorganisms in Foods 7: Microbiological Testing in Food Safety Management.

[11] Ferreira S., Júlio C., Queiroz J.A., Domingues F.C., Oleastro M. (2014). Molecular Diagnosis of *Arcobacter* and *Campylobacter* in Diarrhoeal Samples among Portuguese Patients. Diagn. Microbiol. Infect. Dis..

[12] Ferreira S., Queiroz J.A., Oleastro M., Domingues F.C. (2016). Insights in the Pathogenesis and Resistance of *Arcobacter*: A Review. Crit. Rev. Microbiol..

[13] Chieffi D., Fanelli F., Fusco V. (2020). *Arcobacter butzleri*: Up-to-Date Taxonomy, Ecology, and Pathogenicity of an Emerging Pathogen. Compr. Rev. Food Sci. Food Saf..

[14] Bücker R., Troeger H., Kleer J., Fromm M., Schulzke J.D. (2009). *Arcobacter butzleri* induces barrier dysfunction in intestinal HT-29/B6 cells. J. Infect. Dis..

[15] Flint A., Butcher J., Stintzi A. (2016). Stress Responses, Adaptation, and Virulence of Bacterial Pathogens During Host Gastrointestinal Colonization. Microbiol. Spectr..

[16] Kim S.-H., Chelliah R., Ramakrishnan S.R., Perumal A.S., Bang W.-S., Rubab M., Daliri E.B.-M., Barathikannan K., Elahi F., Park E. (2021). Review on Stress Tolerance in *Campylobacter jejuni*. Front. Cell. Infect. Microbiol..

[17] Poole K. (2012). Bacterial Stress Responses as Determinants of Antimicrobial Resistance. J. Antimicrob. Chemother..

[18] Gundogdu O., da Silva D.T., Mohammad B., Elmi A., Wren B.W., van Vliet A.H.M., Dorrell N. (2016). The *Campylobacter jejuni* Oxidative Stress Regulator RrpB Is Associated with a Genomic Hypervariable Region and Altered Oxidative Stress Resistance. Front. Microbiol..

[19] Kim J.C., Oh E., Hwang S., Ryu S., Jeon B. (2015). Non-Selective Regulation of Peroxide and Superoxide Resistance Genes by PerR in *Campylobacter jejuni*. Front. Microbiol..

[20] Oh E., McMullen L., Jeon B. (2015). Impact of Oxidative Stress Defense on Bacterial Survival and Morphological Change in *Campylobacter jejuni* under Aerobic Conditions. Front. Microbiol..

[21] Gundogdu O., da Silva D.T., Mohammad B., Elmi A., Mills D.C., Wren B.W., Dorrell N. (2015). The *Campylobacter jejuni* MarR-like Transcriptional Regulators RrpA and RrpB Both Influence Bacterial Responses to Oxidative and Aerobic Stresses. Front. Microbiol..

[22] Houf K., Tutenel A., Zutter L., Hoof J.V., Vandamme P. (2000). Development of a Multiplex PCR Assay for the Simultaneous Detection and Identification of *Arcobacter butzleri*, *Arcobacter cryaerophilus* and *Arcobacter skirrowii*. FEMS Microbiol. Lett..

[23] Kern M., Volz J., Simon J. (2011). The Oxidative and Nitrosative Stress Defence Network of *Wolinella succinogenes*: Cytochrome c Nitrite Reductase Mediates the Stress Response to Nitrite, Nitric Oxide, Hhydroxylamine and Hydrogen Peroxide. Environ Microbiol..

[24] Isohanni P., Huehn S., Aho T., Alter T., Lyhs U. (2013). Heat Stress Adaptation Induces Cross-protection Against Lethal Acid Stress Conditions in *Arcobacter butzleri* but not in *Campylobacter jejuni*. Food Microbiol..

[25] Mateus C., Nunes A.R., Oleastro M., Domingues F., Ferreira S. (2021). RND Efflux Systems Contribute to Resistance and Virulence of *Aliarcobacter butzleri*. Antibiotics.

[26] Ferreira S., Correia D.R., Oleastro M., Domingues F.C. (2018). *Arcobacter butzleri* Ciprofloxacin Resistance: Point Mutations in DNA Gyrase A and Role on Fitness Cost. Microb. Drug Resist..

[27] Medina G., Neves P., Flores-Martin S., Manosalva C., Andaur M., Otth C., Lincopan N., Fernández H. (2019). Transcriptional Analysis of Flagellar and Putative Virulence Genes of *Arcobacter butzleri* as an Endocytobiont of Acanthamoeba Castellanii. Arch. Microbiol..

[28] Muyzer G., De Waal E.C., Uitterlinden A.G. (1993). Profiling of Complex Microbial Populations by Denaturing Gradient Gel Electrophoresis Analysis of Polymerase Chain Reaction-Amplified Genes Coding for 16S RRNA. Appl. Environ. Microbiol..

[29] Comtois S.L., Gidley M.D., Kelly D.J. (2003). Role of the Thioredoxin System and the Thiol-Peroxidases Tpx and Bcp in Mediating Resistance to Oxidative and Nitrosative Stress in *Helicobacter pylori*. Microbiology.

[30] Imlay J.A. (2008). Cellular Defenses against Superoxide and Hydrogen Peroxide. Annu Rev Biochem..

[31] Kim J.C., Oh E., Kim J., Jeon B. (2015). Regulation of Oxidative Stress Resistance in *Campylobacter jejuni*, a Microaerophilic Foodborne Pathogen. Front. Microbiol..

[32] Miller W.G., Parker C.T., Rubenfield M., Mendz G.L., Wösten M.M.S.M., Ussery D.W., Stolz J.F., Binnewies T.T., Hallin P.F., Wang G. (2007). The Complete Genome Sequence and Analysis of the Epsilonproteobacterium Arcobacter Butzleri. PLoS ONE.

[33] Atack J.M., Kelly D.J. (2008). Contribution of the stereospecific methionine sulphoxide reductases MsrA and MsrB to oxidative and nitrosative stress resistance in the food-borne pathogen *Campylobacter jejuni*. Microbiology.

[34] Wainwright L.M., Elvers K.T., Park S.F., Poole R.K. (2005). A truncated haemoglobin implicated in oxygen metabolism by the microaerophilic food-borne pathogen *Campylobacter jejuni*. Microbiology.

[35] Hill C., O’Driscoll B., Booth I. (1995). Acid Adaptation and Food Poisoning Microorganisms. Int. J. Food Microbiol..

[36] Chowdhury R., Sahu G.K., Das J. (1996). Stress Response in Pathogenic Bacteria. J. Biosci..

[37] Dressman J.B., Berardi R.R., Dermentzoglou L.C., Russell T.L., Schmaltz S.P., Barnett J.L., Jarvenpaa K.M. (1990). Upper Gastrointestinal (GI) PH in Young, Healthy Men and Women. Pharm. Res..

[38] Cervenka L. (2007). Survival and Inactivation of *Arcobacter* spp., a Current Status and Future Prospect. Crit. Rev. Microbiol..

[39] Murphy C., Carroll C., Jordan K.N. (2003). Induction of an Adaptive Tolerance Response in the Foodborne Pathogen, *Campylobacter jejuni*. FEMS Microbiol. Lett..

[40] Murphy C., Carroll C., Jordan K.N. (2006). Environmental Survival Mechanisms of the Foodborne Pathogen *Campylobacter jejuni*. J. Appl. Microbiol..

[41] Wilson M., Otth L., Aron R., Fernández H. (2010). Susceptibility of *Arcobacter Butzleri* to Human Blood Serum. Arq. Bras. Med. Veterinária e Zootec..

[42] Blaser M.J., Smith P.F., Hopkins J.A., Heinzer I., Bryner J.H., Wang W.L.L. (1987). Pathogenesis of *Campylobacter Fetus* Infections: Serum Resistance Associated with High-Molecular-Weight Surface Proteins. J. Infect. Dis..

[43] Blaser M.J., Perez G.P., Smith P.F., Patton C., Tenover F.C., Lastovica A.J., Wang W.-I.L. (1986). Extraintestinal *Campylobacter jejuni* and *Campylobacter coli* Infections: Host Factors and Strain Characteristics. J. Infect. Dis..

[44] Keo T., Collins J., Kunwar P., Blaser M.J., Iovine N.M. (2011). *Campylobacter* capsule and lipooligosaccharide confer resistance to serum and cationic antimicrobials. Virulence.

[45] Taylor P.W. (1983). Bactericidal and Bacteriolytic Activity of Serum against Gram-Negative Bacteria. Microbiol. Rev..

[46] McManus D.C., David Josephy P. (1995). Superoxide Dismutase Protects Escherichia Coli against Killing by Human Serum. Arch. Biochem. Biophys..

[47] Ginsburg I. (1998). Could Synergistic Interactions among Reactive Oxygen Species, Proteinases, Membrane-Perforating Enzymes, Hydrolases, Microbial Hemolysins and Cytokines Be the Main Cause of Tissue Damage in Infectious and Inflammatory Conditions?. Med. Hypotheses.

[48] Sengupta C., Ray S., Chowdhury R. (2014). Fine Tuning of Virulence Regulatory Pathways in Enteric Bacteria in Response to Varying Bile and Oxygen Concentrations in the Gastrointestinal Tract. Gut Pathog..

[49] Alm R.A., Guerry P., Trust T.J. (1993). The *Campylobacter* Sigma 54 FlaB Flagellin Promoter Is Subject to Environmental Regulation. J. Bacteriol..

[50] Ovesen S., Durack J., Kirk K.F., Nielsen H.L., Nielsen H., Lynch S.V. (2019). Motility and Biofilm Formation of the Emerging Gastrointestinal Pathogen *Campylobacter Concisus* Differs under Microaerophilic and Anaerobic Environments. Gut Microbes.

[51] Marteyn B., Scorza F.B., Sansonetti P.J., Tang C. (2011). Breathing Life into Pathogens: The Influence of Oxygen on Bacterial Virulence and Host Responses in the Gastrointestinal Tract. Cell. Microbiol..

[52] Ho H.T.K., Lipman L.J.A., Wösten M.M.S.M., Van Asten A.J.A.M., Gaastra W. (2008). *Arcobacter* spp. Possess Two Very Short Flagellins of Which FlaA Is Essential for Motility. FEMS Immunol. Med. Microbiol..

[53] Magana M., Sereti C., Ioannidis A., Mitchell C.A., Ball A.R., Magiorkinis E., Chatzipanagiotou S., Hamblin M.R., Hadjifrangiskou M., Tegos G.P. (2018). Options and Limitations in Clinical Investigation of Bacterial Biofilms. Clin. Microbiol. Rev..

[54] Adetunji V.O., Adedeji A.O., Kwaga J. (2014). Assessment of the Contamination Potentials of Some Foodborne Bacteria in Biofilms for Food Products. Asian Pac. J. Trop. Med..

[55] Costerton J.W., Stewart P.S., Greenberg E.P. (1999). Bacterial Biofilms: A Common Cause of Persistent Infections. Science.

[56] Ferreira S., Fraqueza M.J., Queiroz J.A., Domingues F.C., Oleastro M. (2013). Genetic Diversity, Antibiotic Resistance and Biofilm-Forming Ability of *Arcobacter Butzleri* Isolated from Poultry and Environment from a Portuguese Slaughterhouse. Int. J. Food Microbiol..

[57] Šilha D., Sirotková S., Švarcová K., Hofmeisterová L., Koryčanová K., Šilhová L. (2021). Biofilm Formation Ability of Arcobacter-like and *Campylobacter* Strains under Different Conditions and on Food Processing Materials. Microorganisms.

[58] Girbau C., Martinez-Malaxetxebarria I., Muruaga G., Carmona S., Alonso R., Fernandez-Astorga A. (2017). Study of Biofilm Formation Ability of Foodborne Arcobacter Butzleri under Different Conditions. J. Food Prot..

[59] Rather M.A., Gupta K., Mandal M. (2021). Microbial Biofilm_ Formation, Architecture, Antibiotic Resistance, and Control Strategies.Pdf. Brazilian J. Microbiol..

[60] Švarcová K., Hofmeisterová L., Švecová B., Šilha D. (2022). In Vitro Activity of Water Extracts of Olive Oil against Planktonic Cells and Biofilm Formation of Arcobacter-like Species.Pdf. Molecules.

[61] Švarcová K., Pejchalová M., Šilha D. (2022). The Effect of Antibiotics on Planktonic Cells and Biofilm Formation Ability of Collected Arcobacter-like Strains and Strains Isolated within the Czech Republic. Antibiotics.

[62] Chaban B., Hughes H.V., Beeby M. (2015). The Flagellum in Bacterial Pathogens: For Motility and a Whole Lot More. Semin. Cell Dev. Biol..

[63] Pizarro-Cerdá J., Cossart P. (2006). Bacterial Adhesion and Entry into Host Cells. Cell.

[64] Cossart P., Sansonetti P.J. (2004). Bacterial Invasion: The Paradigms of Enteroinvasive Pathogens. Science.

[65] Schönknecht A., Alter T., Gölz G. (2020). Detection of Arcobacter Species in Different Intestinal Compartments of Broiler Chicken during Slaughter and Processing. Microbiologyopen.

[66] Ho H.T.K., Lipman L.J.A., Hendriks H.G.C.J.M., Tooten P.C.J., Ultee T., Gaastra W. (2007). Interaction of *Arcobacter* spp. with Human and Porcine Intestinal Epithelial Cells. FEMS Immunol. Med. Microbiol..

[67] Buzzanca D., Botta C., Ferrocino I., Alessandria V., Houf K., Rantsiou K. (2021). Functional pangenome analysis reveals high virulence plasticity of *Aliarcobacter butzleri* and affinity to human mucus. Genomics.

[68] Koolman L., Whyte P., Burgess C., Bolton D. (2016). Virulence gene expression, adhesion and invasion of *Campylobacter jejuni* exposed to oxidative stress (H_2_O_2_). Int. J. Food Microbiol..

